# Increased oxidative stress mediates the antitumor effect of PARP inhibition in ovarian cancer

**DOI:** 10.1016/j.redox.2018.03.016

**Published:** 2018-03-30

**Authors:** Dong Hou, Zhaojian Liu, Xiuhua Xu, Qiao Liu, Xiyu Zhang, Beihua Kong, Jian-Jun Wei, Yaoqin Gong, Changshun Shao

**Affiliations:** a Key Laboratory of Experimental Teratology, Ministry of Education/Department of Molecular Medicine and Genetics, Shandong University School of Medicine, Jinan, Shandong 250012, China; b Department of Cell Biology, Shandong University School of Medicine, Jinan, Shandong 250012, China; c Department of Obstetrics and Gynecology, Qilu Hospital of Shandong University, Jinan, Shandong 250012, China; d Department of Pathology, Northwestern University School of Medicine, Chicago, IL, USA; e The First Affiliated Hospital of Soochow University and State Key Laboratory of Radiation Medicine and Protection, Institutes for Translational Medicine, Soochow University, 199 Ren Ai Road, Suzhou, Jiangsu 215123, China

**Keywords:** PARP1, Oxidative stress, NADPH oxidases, Ovarian cancer

## Abstract

PARP inhibitors have been widely tested in clinical trials, especially for the treatment of breast cancer and ovarian cancer, and were shown to be highly successful. Because PARP primarily functions in sensing and repairing DNA strand breaks, the therapeutic effect of PARP inhibition is generally believed to be attributed to impaired DNA repair. We here report that oxidative stress is also increased by PARP inhibition and mediates the antitumor effect. We showed that PARP1 is highly expressed in specimens of high grade serous ovarian carcinoma and its activity is required for unperturbed proliferation of ovarian cancer cells. Inhibition or depletion of PARP leads to not only an increase in DNA damage, but also an elevation in the levels of reactive oxygen species (ROS). Importantly, antioxidant N-acetylcysteine (NAC) significantly attenuated the induction of DNA damage and the perturbation of proliferation by PARP inhibition or depletion. We further showed that NADPH oxidases 1 and 4 were significantly upregulated by PARP inhibition and were partially responsible for the induction of oxidative stress. Depletion of NOX1 and NOX4 partially rescued the growth inhibition of PARP1-deficient tumor xenografts. Our findings suggest that in addition to compromising the repair of DNA damage, PARP inhibition or depletion may exert extra antitumor effect by elevating oxidative stress in ovarian cancer cells.

## Highlights

• PARP1 is overexpressed in ovarian cancer.
• PARP inhibition increases oxidative stress and oxidative DNA damage.
• PARP inhibition increases ROS by upregulating NOX1 and NOX4.
• Oxidative stress mediates the antitumor effect of PARP inhibition.

## Graphical abstract

**Figure:**
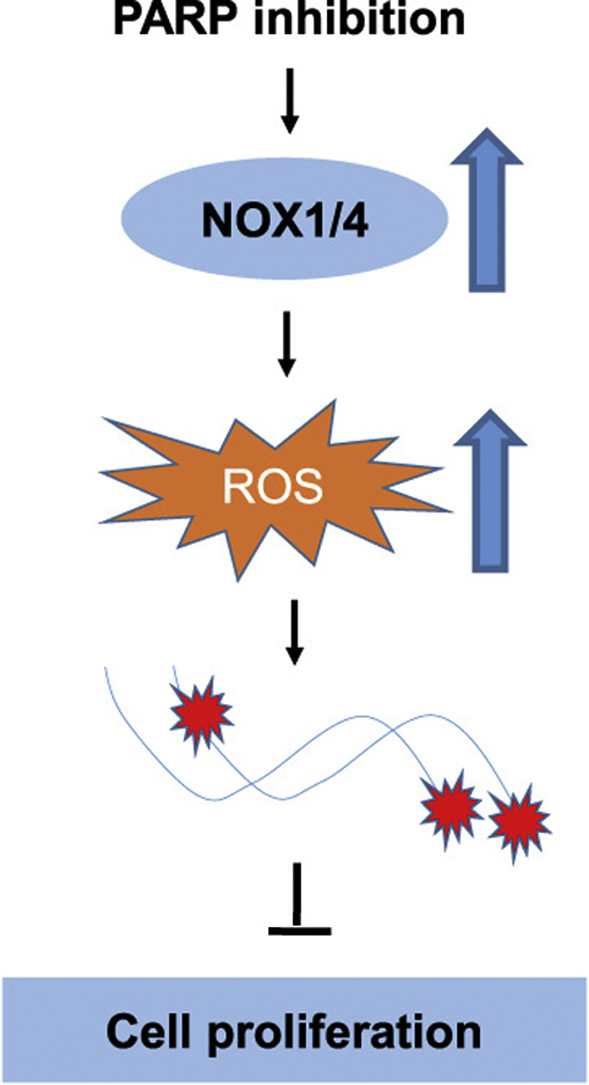

## 1 Introduction

Due to metabolic and signaling aberrations, cancer cells usually have high levels of reactive oxygen species (ROS), which further drive cancer progression by inducing mutations and activating oncogenic pathways [1]. However, excessive production of ROS may also lead to cell death or senescence, and cancer cells generally acquire and rely on a high antioxidant capacity to offset the detrimental effects of the high output of ROS. Therefore, therapeutic strategies that were designed to disrupt the antioxidant defense system in cancer are being actively pursued. Excessive production of ROS will cause various types of DNA damage, including base damage, single-strand breaks (SSBs) and double-strand breaks (DSBs) [2], [3]. Base excision repair (BER) plays a critical role in the repair of oxidative base damage and SSBs, whereas homologous recombination repair (HRR) and non-homologous end joining (NHEJ) are essential for the repair of DSBs. Some of those DNA repair pathways are also upregulated in cancer and contribute to the progression of malignancy [4]. PARP1, a protein that senses DNA strand breaks and orchestrates their repair, plays an important role in the cellular response to oxidative DNA damage [4], [5], [6]. However, in response to excessive oxidative stress, persistent PARP1 hyperactivation may lead to cell death [5], [7]. PARP1 hyperactivation has also been shown to occur when DNA repair is defective, as in XPA-deficient cells, XRCC1 mutant individuals and in HRR-defective cancer cells [8], [9], [10].

Cancer cells lacking functional BRCA1 or BRCA2, critical players in HRR, were found to be particularly sensitive to PARP1 inhibition [11], [12]. Cells with defective HRR are generally associated with PARP hyperactivation [8]. It was generally believed that when the repair of SSBs was blocked by PARP1 inhibition, SSBs would be converted into DSBs in S-phase that can only be repaired by HRR, therefore impaired HRR, as in cancer cells carrying BRCA1 or BRCA2 mutations, would render synthetic lethality with PARP1 inhibition [13], [14].

Ovarian cancer is the most lethal gynecological cancer. It is heterogeneous in histological origin, but high grade serous carcinoma, which originates from fallopian tube epithelial cells, accounts for majority of the cases and most of the lethality [15]. Because of lack of symptoms and biomarkers at early stage, most of the ovarian cancer cases are already progressed to advanced stages when diagnosed. Ovarian cancer is usually managed by surgical resection followed by platinum-based chemotherapy [16]. The high response rate of ovarian cancer to platinum analogues is believed to be due to a high prevalence of defective homologous recombination repair [17]. In recent years, PARP inhibitors have been studied in various clinical trials, especially for cancers with defective HRR [18]. However, the mechanisms underlying the synthetic lethality between PARP inhibition and defective HRR have not been fully elucidated [17]. A recent study showed that PARP inhibitor niraparib was also effective against HRR-proficient ovarian cancer, albeit to a lesser extent when compared to HRR-deficient cancer [18]. Therefore, how PARP inhibitors exert their therapeutic effects on cancer remains to be further investigated.

In this report we studied the role of PARP1 in the proliferation of ovarian cancer cells. We observed that PARP1 is overexpressed in high-grade serous ovarian carcinoma when compared to fallopian tubes and PARP1 inhibition greatly reduced the proliferation of cancer cells. Importantly, we found that the antitumor effect of PARP1 inhibition is attributable to increased oxidative stress that is partially mediated by the upregulation of NADPH oxidases NOX1 and NOX4. Pharmacological inhibition or depletion of NOX1 and NOX4 significantly attenuated the antitumor effect of PARP1 inhibition.

## 2 Results

### 2.1 PARP1 is overexpressed in ovarian cancer

**Fig. 1:**
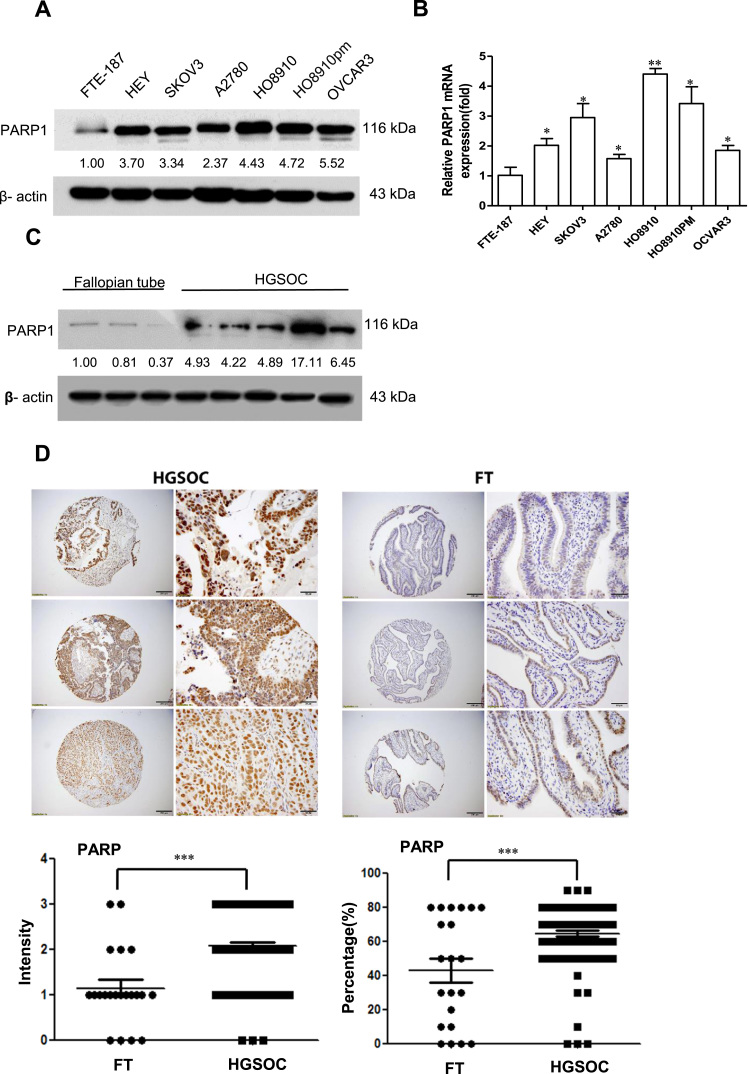
PARP1 is overexpressed in ovarian cancer. (A and B) Western blotting and RT-PCR analysis of PARP1 expression in immortalized fallopian tube epithelial cell line (FTE-187) and ovarian cancer cell lines. (C) Western blotting analysis of PARP1 protein expression in ovarian cancer and fallopian tube tissues. (D) Immunohistochemistry staining of PARP1 expression in high-grade serous ovarian carcinoma (HGSOC) and fallopian tube tissues (FT).The numbers shown below bands were folds of band intensities relative to control. Band intensities were quantified by ImageJ and normalized to β-actin. Each experiment was repeated three times.*p< 0.05, * *p< 0.01,*** p < 0.001 (compared to FTE-187 or FT, *t*-test).

PARP1 was measured by Western blot in several ovarian cancer cell lines and FTE-187, immortalized fallopian tube epithelial cells. As shown in Fig. 1A, PARP1 levels are generally higher in cancer cells than in FTE-187 cells. PARP1 mRNA followed a similar trend (Fig. 1B). Western blot analysis of proteins extracted from high-grade serous ovarian cancer (HGSOC) and fallopian tubes (FT) showed that PARP1 levels were much higher in HGSOC tissues than in FT (Fig. 1C). We further analyzed the expression levels of PARP1 in 94 specimens of HGSOC and 26 matched FT tissues. As shown in Fig. 1D, PARP1 levels are significantly higher in HGSOC when compared to FT tissues. These results indicate that PARP1 is generally overexpressed in ovarian cancer.

### 2.2 PARP1 inhibition leads to decreased proliferation of ovarian cancer cells

**Fig. 2:**
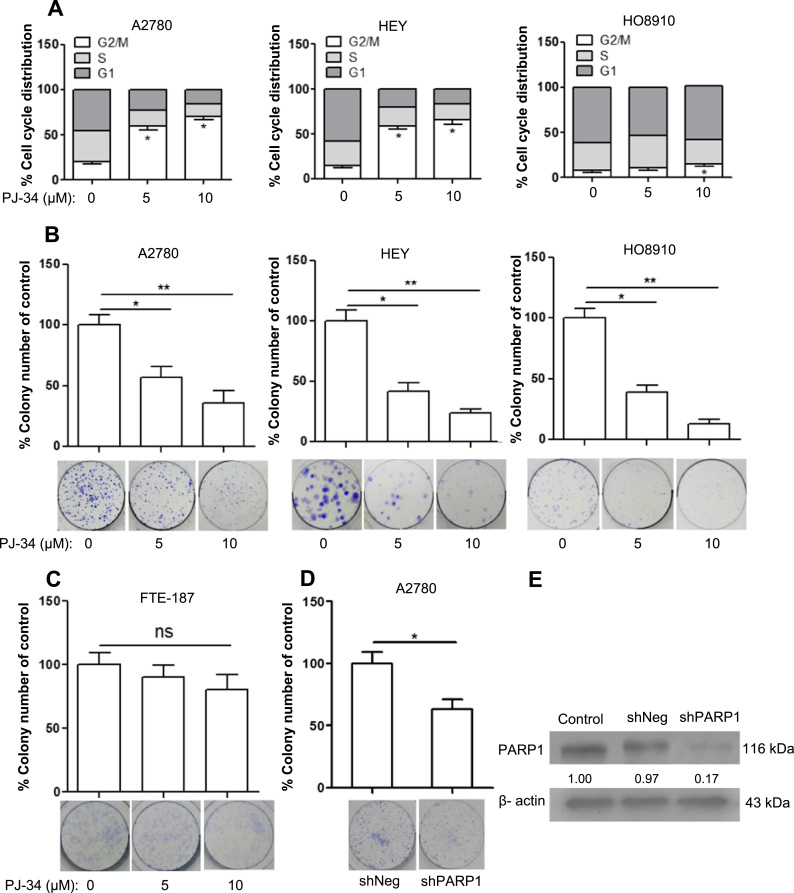
PARP1 inhibition reduces proliferation of ovarian cancer cells. (A) Cell cycle distribution of A2780, HO8910 and HEY cells after being treated with PJ-34 for 48 h. The cell cycle distribution was measured by flow cytometry. (B) Colony-forming efficiency of ovarian cancer cells after treatment with PJ-34 for two weeks. (C) Colony-forming efficiency of FTE-187 after treated by different concentration of PJ-34 for two weeks. (D) Colony-forming efficiency of A2780 shPARP1 cells and A2780 shNeg cells. (E) RNAi efficiency was evaluated by Western blotting. The numbers shown below bands were folds of band intensities relative to control. Band intensities were quantified by ImageJ and normalized to β-actin. Each experiment was repeated three times.*p< 0.05, **p< 0.01 (compared to control, *t*-test).

To determine whether PARP1 overexpression contributes to the survival and/or proliferation of ovarian cancer cells, we applied PJ-34 to cultured cancer cells, A2780, HEY and HO8910, and evaluated apoptosis and cell cycle progression. As shown in Fig S1A, no increase in apoptosis was detected after PJ-34 treatment for 48 h. However, cells treated with PJ-34 for 48 h showed an increased accumulation at G2 phase (Fig. 2A). This result suggested that PARP1 inhibition led to decreased proliferation. This was confirmed by colony formation assay. As shown in Fig. 2B, colony formation was greatly reduced by PJ-34. Colony formation of FTE-187 cells, immortalized fallopian tube epithelial cells, was only mildly reduced (statistically not significant) (Fig. 2C). We also stably knocked down PARP1 in A2780 cells and analyzed their colony formation. As shown in Fig. 2D and 2E, shPARP1 cells were significantly reduced in their colony formation when compared to control cells (shNeg).

### 2.3 PARP1 inhibition leads to increased oxidative stress and oxidative DNA damage

**Fig. 3:**
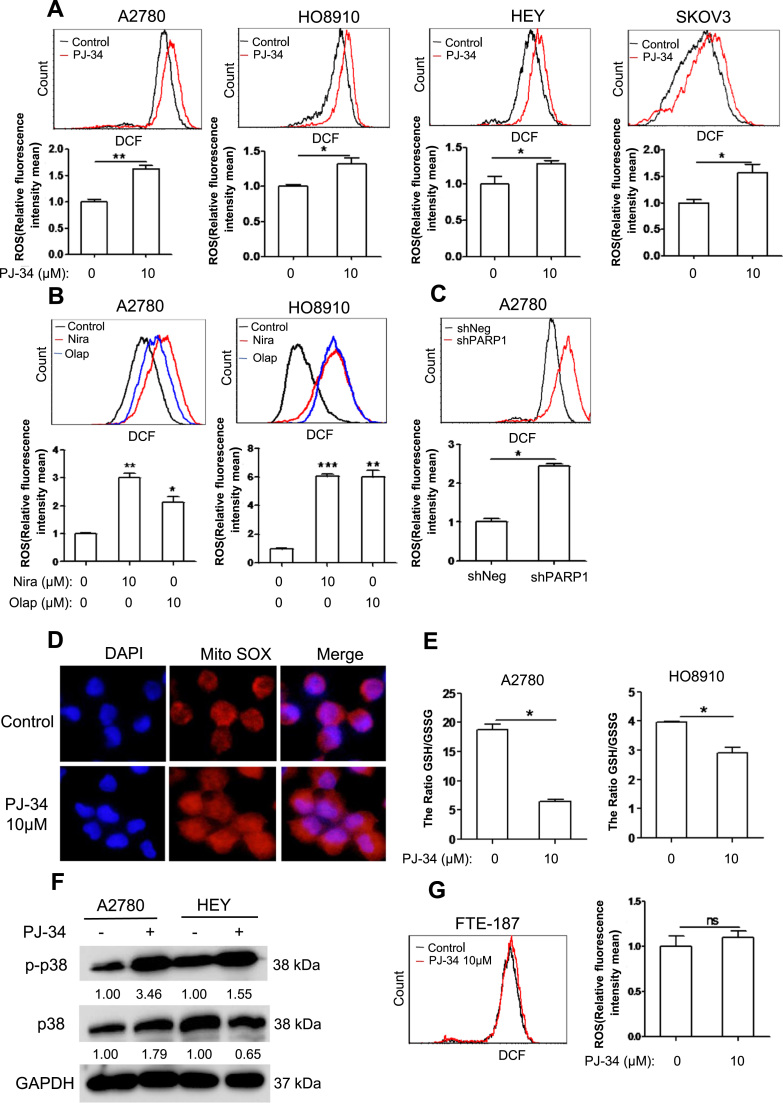
PARP1 inhibition increases the ROS levels of ovarian cancer cells. (A) ROS distribution measured by flow cytometry in A2780, HO8910, HEY and SKOV3 cells treated with 10 µM PJ-34 for 48 h. (B) ROS distribution measured by flow cytometry in A2780 and HO8910 treated with 10 µM Niraparib or Olaparib for 48 h. (C) ROS distribution measured by flow cytometry in A2780 depletion of PARP1. (D) Fluorescence staining of MitoSOX in A2780 treated with 10μM PJ-34. (E) The GSH/GSSG ratio was measured by a GSH and GSSG assay kit in A2780 and HO8910 treated with different concentration of PJ-34 for 48 h. (F) Western blotting analysis of p-p38 and p38 protein expression in A2780 and HEY treated with 10 μM PJ-34 for 48 h. (G) ROS distribution measured by flow cytometry in FTE-187 treated with different concentration of PJ-34 for 48 h. The numbers shown below bands were folds of band intensities relative to control. Band intensities were quantified by ImageJ and normalized to GAPDH. Each experiment was repeated three times.*p< 0.05, **p< 0.01, ***p< 0.001 (compared to control, *t*-test).

Ovarian cancer cells rely on high level of reactive oxygen species for their proliferation [19], [20], and PARP hyperactivation was known to induce ROS [21]. We next tested whether the impairment in proliferation by PJ-34 was due to changes in ROS. Surprisingly, PARP inhibition led to a significant elevation in ROS in all four cell lines tested (Fig. 3A). Two additional PARP1 inhibitors, niraparib and oliparib, were observed to have a similar effect on A2780 and HO8910 cells (Fig. 3B). Depletion of PARP1 also led to an elevation in ROS (Fig. 3C). The level of superoxide, as measured by MitoSox, was increased by PARP inhibition (Fig. 3D). The ratio of GSH/GSSG was also reduced by PARP inhibition (Fig. 3E). Phosphorylation of p38MAPK, which usually occurs in response to oxidative stress, was also increased in cancer cells when PARP1 was inhibited (Fig. 3F). Of note, PARP inhibition had no effect on the level of ROS in FTE-187 cells (Fig. 3G).

**Fig. 4:**
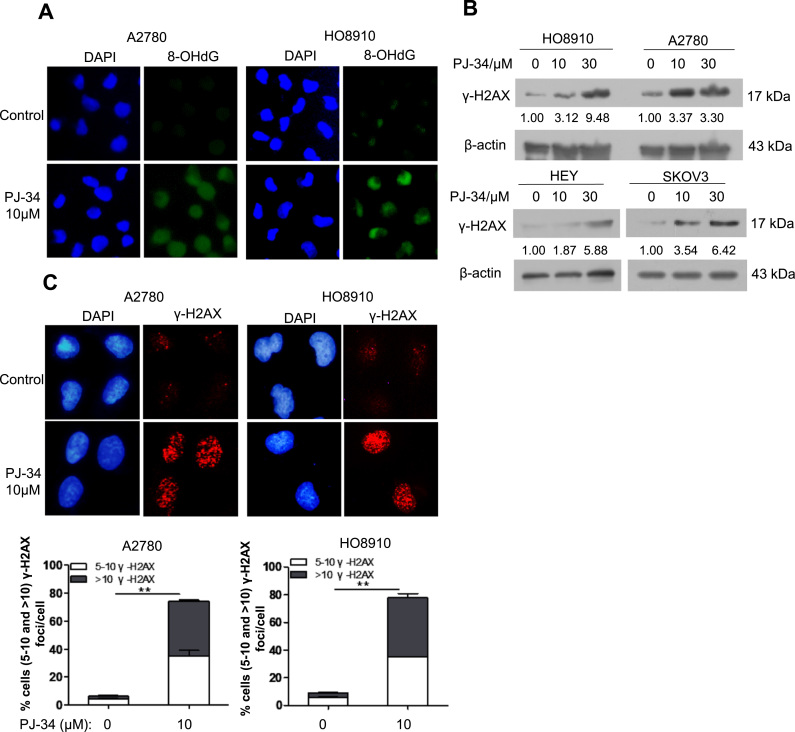
PARP1 inhibition increases oxidative DNA damage in ovarian cancer cells. (A) Immunofluorescence staining of 8-OHdG in A2780 and HO8910 treated with 10 μM PJ-34 for 48 h. (B) Western blotting analysis of γ-H2AX protein expression in HO8910, A2780, HEY, and SKOV3 treated with different concentration of PJ-34 for 48 h. (C) Immunofluorescence staining of γ-H2AX in A2780 and HO8910 treated with 10 μM PJ-34 for 48 h. The numbers shown below bands were folds of band intensities relative to control. Band intensities were quantified by ImageJ and normalized to β-actin.Each experiment was repeated three times. **p< 0.01 (compared to control, *t*-test).

We next examined the level of oxidative base damage in PJ-34 treated ovarian cancer cells. As shown in Fig. 4A, there was a significant increase in staining intensity of 8-oxo-dG, a marker of oxidative base damage. PARP inhibition is known to cause DSBs independent of BRCA1 or BRCA2 function [12]. We therefore examined the levels of γ-H2AX in PJ-34 treated ovarian cancer cells. Indeed, increased levels of γ-H2AX were detected by both Western blot and immunofluorescence staining in PJ-34 treated cells (Fig. 4B and 4C).

To determine whether the induction of oxidative stress by PARP inhibition also applies to other types of cancer cells, we subjected breast cancer cells MCF-7, fibrosarcoma cells HT1080, lung cancer cells A549 and colorectal cancer cells HCT116 to PJ-34. As shown in Fig S2, an increase in ROS level was detected only in MCF-7 cells.

### 2.4 Antioxidant rescues the antiproliferative effect of PARP inhibition or depletion

**Fig. 5:**
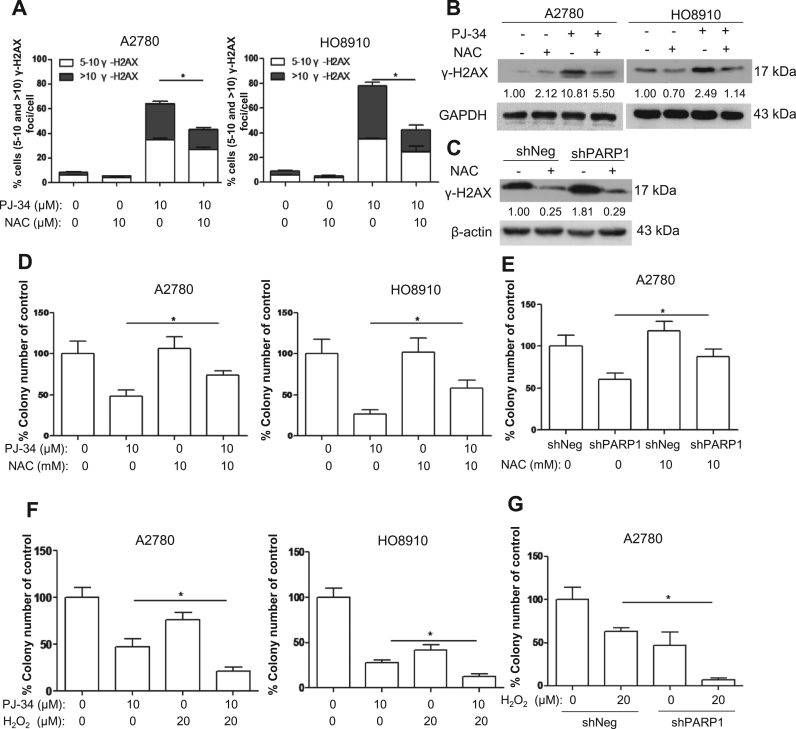
Antioxidant reduces oxidative DNA damage and rescues clonogenic survival in PARP1-inhibited cells. (A) Scoring of γ-H2AX foci in A2780 and HO8910 treated with 10 μM PJ-34 alone or in combination with 10 mM NAC for 48 h. (B) Western blotting analysis of γ-H2AX levels in A2780 and HO8910 treated with 10 μM PJ-34 alone or in combination with 10 mM NAC for 48 h. (C) Western blotting analysis of γ-H2AX levels in shPARP1 and shNeg (control) A2780 cells treated with 10 mM NAC for 48 h. (D) Clonogenic assay of A2780 and HO8910 cells treated with 10 μM PJ-34 alone or in combination with 10 mM NAC. (E) Clonogenic assay of A2780 shNeg and shPARP1 cells treated with 10 mM NAC. (F) Clonogenic assay of A2780 and HO8910 cells treated with 10 μM PJ-34 alone or in combination with 20 μM H_2_O_2_. (G) Clonogenic assay of A2780 shNeg and shPARP1 cells treated with 20 μM H_2_O_2_. The numbers shown below bands were folds of band intensities relative to control. Band intensities were quantified by ImageJ and normalized to β-actin or GAPDH. Each experiment was repeated three times. *p< 0.05 (one-way ANOVA or *t*-test).

PARP1 is involved in many biological processes including the repair of base damage and SSBs [4], [5]. It is possible that the antiproliferative effect exerted by PARP1 inhibition is due to impaired DNA repair or other processes other than oxidative stress. We therefore tested whether antioxidant could rescue the antiproliferative effect of PARP inhibition. As shown in Fig. 5A-C, the levels of DSBs induced by PARP inhibition or depletion were significantly reduced by N-acetylcysteine (NAC). Importantly, colony formation was significantly rescued by NAC (Fig. 5D and 5E).

If oxidative stress mediates the antiproliferative effect of PARP inhibition on ovarian cancer cells, PARP1 inhibition or depletion would be expected to render ovarian cancer cells more sensitive to exogenous H_2_O_2._ Indeed, H_2_O_2_ caused a more pronounced decrease in colony formation when PARP1 was inhibited or depleted (Fig. 5F and 5G). These results suggest that oxidative stress plays an important role in mediating the antiproliferative effect of PARP inhibition or depletion.

### 2.5 Upregulation of NADPH oxidases is responsible for ROS induction by PARP inhibition

We next explored the possible mechanisms by which ROS are increased by PARP inhibition. We first tested if PARP1 plays any role in the function of NRF2, a master regulator of antioxidant defense. It was reported that PARP1 is structurally required for the proper function of NRF2 [22]. We examined several NRF2 target genes but found no difference in expression when cells were treated with PJ-34 (Fig S3A), although PARP1 depletion downregulated NRF2 target genes modestly (Fig S3B), which is consistent with the previous report [22]. Because p38 was greatly activated by PARP inhibition and p38 activation could mediate an increase in ROS [23], we wondered if p38 activation could be responsible for ROS increase. However, p38 inhibitor was unable to block the increase in ROS (Fig S3C), which ruled out p38 activation as a mediator of the ROS increase.

**Fig. 6:**
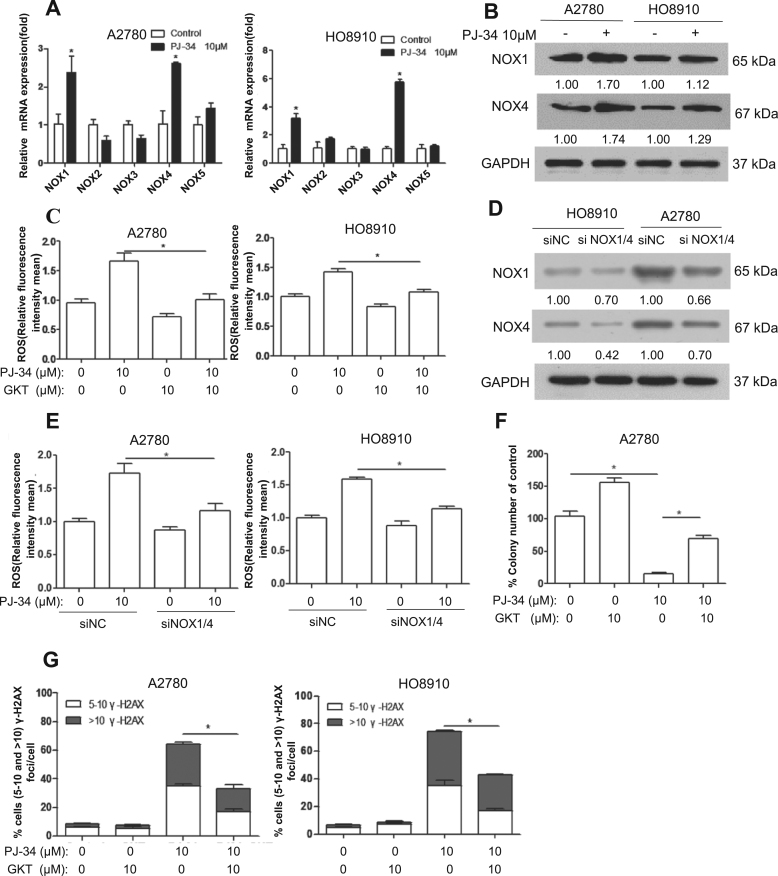
Inhibition of PARP1 leads to upregulation of NOX1 and NOX4. (A) RT-PCR analysis of various human NOX genes. A2780 and HO8910 cells were treated with 10 μM PJ-34, the expression levels of NOX mRNAs were determined using real-time RT-PCR. (B) Western blotting analysis of NOX1 and NOX4 protein expression in A2780 and HO8910 treated with 10 μM PJ-34 for 48 h. (C) ROS distribution measured by flow cytometry in A2780 and HO8910 treated with 10 μM PJ-34 alone or in combination with 10 mM GKT for 48 h. (D and E) A2780 and HO8910 were transfected with mock siRNA or NOX1/4, (D) Western blotting analysis of NOX1 and NOX4 protein expression, (E) ROS distribution measured by flow cytometry. Cells were treated with 10 μM PJ-34 for 48 h. (F) Clonogenic assay showed inhibitory effect of 10 μM PJ-34 alone or in combination with 10 mΜ GKT in A2780. (G) Scoring of γ-H2AX foci in A2780 and HO8910 treated with 10 μM PJ-34 alone or in combination with 10 mM GKT for 48 h. The numbers shown below bands were folds of band intensities relative to control. Band intensities were quantified by ImageJ and normalized to GAPDH. Each experiment was repeated three times. *p< 0.05 (one-way ANOVA or *t*-test).

NADPH oxidases (NOX) are a major source of ROS in cancer [24]. We next determined whether the expression of NOX family members was altered by PARP inhibition. As shown in Fig. 6A, the levels of NOX1 and NOX4 transcripts were significantly increased by PJ-34 treatment, in both A2780 and HO8910 cells. The upregulation of NOX1 and NOX4 was confirmed at protein level (Fig. 6B). Importantly, GKT137831 (GKT), a specific inhibitor of NOX1 and NOX4, significantly attenuated the increase in ROS caused by PJ-34 (Fig. 6C). Similarly, simultaneous depletion of NOX1 and NOX4 also reduced the ROS level in cancer cells treated with PJ-34 (Fig. 6D and 6E). The reduction in colony formation caused by PJ-34 was also rescued by GKT treatment (Fig. 6F). The levels of DSBs induced by PARP inhibition were attenuated by GKT (Fig. 6G). Together, these results suggest that upregulation of NOX1 and NOX4 may mediate the elevation of ROS when PARP is inhibited.

### 2.6 Suppression of tumor growth by PARP inhibition in vivo is accompanied by upregulation of NADPH oxidases

**Fig. 7:**
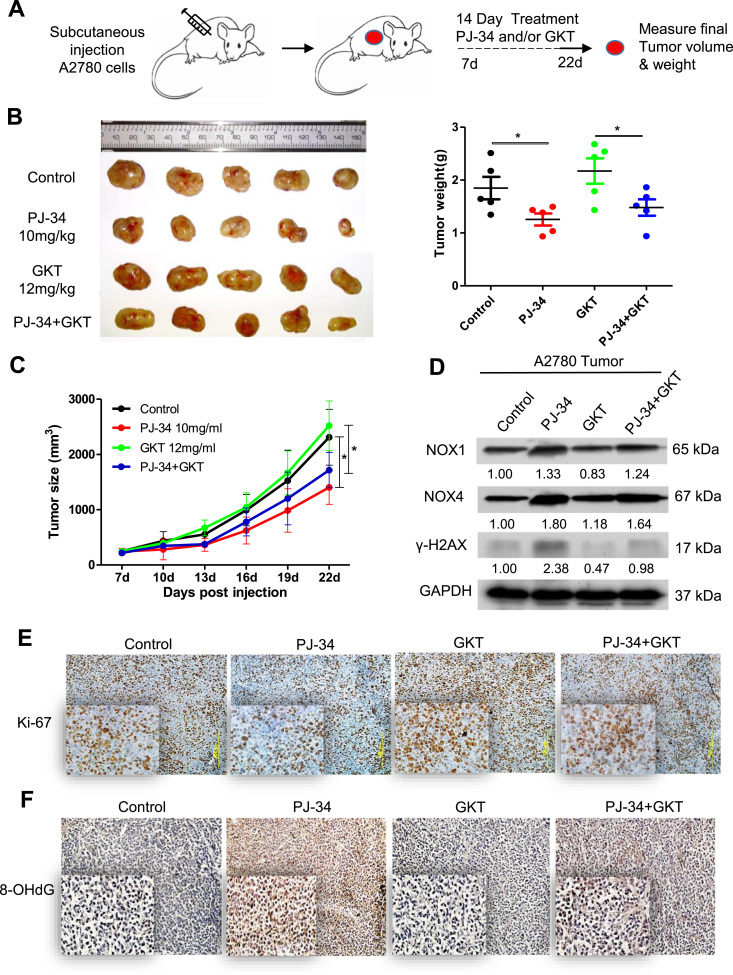
PARP Inhibition impedes tumor growth in vivo. (A) Scheme for the subcutaneous xenografts under 14-day treatment paradigm. Mice were randomized into one of four treatment groups; vehicle only (DMSO; n = 6), 10 mg/kg PJ-34 only (PARPi; n = 6), GKT only (NOX1/4i; n = 6) and 10 mg/kg PJ-34 plus 12 mg/kg GKT (n = 6)(given daily by intraperitoneal injection). Tumors volumes were measured every 3 days and final weights were taken at day 22. (B) Left, images of A2780 tumors for each treatment group.Right, tumor weights at day 22. (C) Growth curves of tumors from transplanted A2780 cells in nude mice. (D) Western blotting analysis of NOX1 and NOX4 protein expression in A2780 tumors for each treatment group. Experiment was repeated three times. (E and F) Representative images of immunohistochemistry staining using Ki-67 and 8-OHdG antibody in A2780 tumors for each treatment group. The numbers shown below bands were folds of band intensities relative to control. Band intensities were quantified by ImageJ and normalized to GAPDH. *p< 0.05 (one-way ANOVA or *t*-test).

We next tested whether PJ-34 could inhibit tumor growth in vivo. To this end, A2780 cells were subcutaneously transplanted into flanks of nude mice, seven days later the tumor-bearing mice were randomly divided into four groups and were treated with vehicle, PJ-34, GKT and PJ-34+GKT, respectively (Fig. 7A). PJ-34 significantly inhibited the growth of tumor xenografts when compared to the control (Fig. 7B and 7C). Consistent with the in vitro studies (Fig. 6B), NOX1 and NOX4 were greatly upregulated in tumor xenografts treated with PJ-34 (Fig. 7D). In keeping with the antiproliferative effect of PJ-34 on cancer cells in vitro, there was a significant decrease in the abundance of Ki-67 positive cells in the tumor xenografts (Fig. 7E). Oxidative base damage, as reflected by the staining for 8-oxo-dG, was greatly increased in the tumors treated with PJ-34 (Fig. 7F). While GKT significantly neutralized the antiproliferative effect of PJ-34 and alleviated the associated oxidative DNA damage (Fig. 7E and 7F), the rescuing effect of GKT on tumor growth was only suggestive, with no statistical significance (Fig. 7B).

### 2.7 Depletion of NADPH oxidases rescues the growth inhibition of PARP1-deficient tumor xenografts

**Fig. 8:**
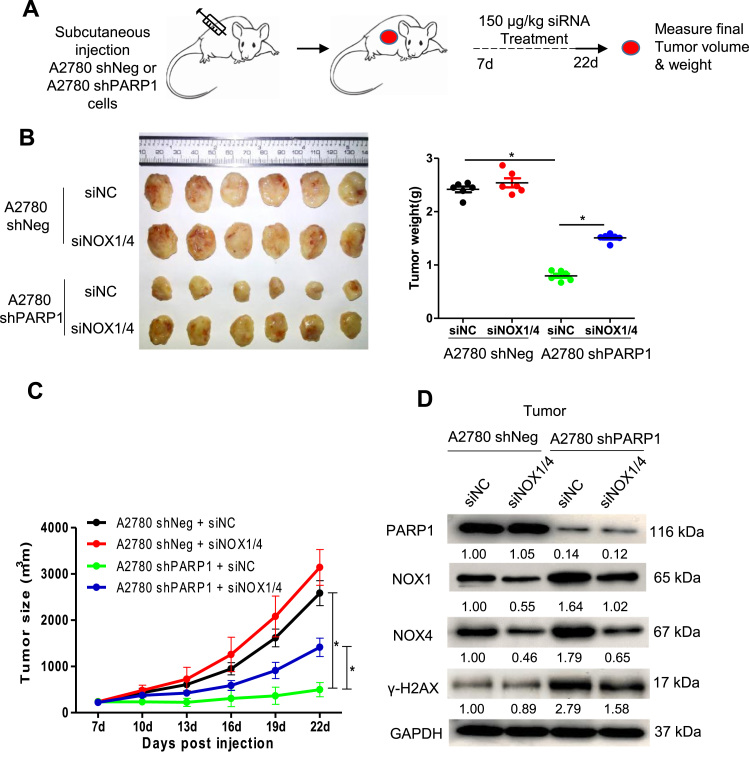
Depletion of NOX1 and NOX4 rescues growth of PARP1-depleted tumors. (A) Schematic diagram for the treatment paradigm. Mice were randomized into one of four groups and were treated as indicated. Tumors volumes were measured every 3 days and final weights were taken at day 22. (B) Left, images of A2780 shNeg and A2780 shPARP1 tumors for each treatment group. Right, tumor weights at day 22. (C) Growth curves of tumors from transplanted A2780 shNeg and A2780 shPARP1 cells in nude mice. (D) Western blotting analysis of NOX1 and NOX4 protein expression in tumors for each treatment group. Experiment was repeated three times. The numbers shown below bands were folds of band intensities relative to control. Band intensities were quantified by Image J and normalized to GAPDH. *p< 0.05 (one-way ANOVA or *t*-test).

Because the PARP1-depletion resulted in ROS elevation to a greater extent than the PJ-34 treatment in A2780 cells (Fig. 3C), we next transplanted A2780-shPARP1 and control cells into nude mice and, seven days later, subjected the mice to intravenous injection (i.v.) of liposomal siRNA targeting NOX1 and NOX4 or non-silencing siRNA (Fig. 8A). As expected, the tumor xenografts formed by A2780-shPARP1 cells were smaller than those by A2780-shNeg cells. Importantly, depletion of the two NADPH oxidases significantly rescued the growth-inhibitory effect of PARP deletion (Fig. 8B and 8C). The depletion of NOX1 and NOX4 in tumor xenografts was confirmed by Western blot (Fig. 8D). As expected, depletion of the NADPH oxidases attenuated DNA damage, as reflected by γ-H2AX, in tumor xenografts formed by A2780-shPARP1 cells (Fig. 8D). It appears that the rescuing effect of NOX inhibition or depletion may manifest when tumor growth is more drastically inhibited by PARP1 inhibition or depletion.

## 3 Discussion

High expression of PARP1 in ovarian cancer is associated with poor survival [25]. In recent years, PARP inhibitors have been studied in various clinical trials, especially for cancers with defective HRR [18]. PARP inhibitor niraparib was also shown to be effective against HRR-proficient ovarian cancer, albeit to a lesser extent when compared to HRR-deficient cancer [18]. Therefore, PARP inhibitors may also exert their cytotoxic effects on cancer cells independent of HRR. The findings we presented in this study indicate that in addition to compromising DNA repair, PARP inhibitors also elevate oxidative stress and induce oxidative DNA damage in cancer cells. PARP inhibition or depletion also renders cancer cells more sensitive to H_2_O_2_. Because PARP is essential in repair of oxidative DNA damage, it can be argued that the increased persistence of oxidative DNA damage when PARP is inhibited may be caused by a failure to repair the damage. However, ROS levels were increased by PARP inhibitors in several ovarian cancer cell lines. In addition, we found that NOX1 and NOX4 were upregulated by PARP inhibition and were responsible for the elevation of ROS and the consequent cytotoxicity. Therefore, increased oxidative stress should also be regarded as an important mediator of the cytotoxic effect of PARP inhibition. We recently reported that berberine can render ovarian cancer cells more sensitive to PARP inhibition by inducing oxidative stress and downregulating HRR [26]. While defective HRR is recognized to be an indicator for PARP inhibition therapy, it appears that oxidative stress and oxidative DNA damage in ovarian cancer cells may also be considered as potential markers for PARP inhibition-based therapy.

In response to PARP inhibition or depletion, NOX1 and NOX4 were significantly upregulated in vitro and in vivo, and GKT137831, a specific inhibitor of NOX1 and NOX4, effectively attenuated oxidative DNA damage and rescued the perturbed proliferation, when measured by clonogenic assay in vitro and by immunostaining of Ki-67 of tumor xenograft in vivo. However, the rescuing effect of GKT137831 on tumor size reduction caused by PJ-34 was only suggestive. The less pronounced tumor reduction by PJ-34 may have made the rescuing effect less obvious. When using cancer cells with stable knockdown of PARP1, which form much smaller tumors, a significant rescuing effect by NOX1/4 depletion was detected. The difference between tumor xenografts formed by PARP1-depleted and proficient ovarian cancer cells is more pronounced. Correspondingly, depletion of NOX1 and NOX4 significantly rescued the growth inhibition of tumors formed by PARP1-depleted ovarian cancer cells. This result indicates that the upregulation of NOX1 and NOX4 is partially responsible for the tumor-inhibitory effect of PARP1 depletion in vivo.

It should be noted that PARP inhibition does not lead to an increase in ROS level in some other cancer types (Fig S2). Ovarian cancer is particularly known for possessing a robust antioxidant defense that is mediated by the upregulation of NRF2 function [27]. This antioxidant defense system appears to be required for sustaining the survival and the malignant phenotypes of ovarian cancer cells [28]. In addition to the upregulation of NRF2 antioxidant function, repression of NADPH oxidases, an important source of ROS, by PARP1 may also contribute to the maintenance of redox homeostasis in ovarian cancer cells. We may speculate that for cancer cells that do not experience oxidative stress, repression of NADPH oxidases may not be critical, or even undesirable, for cancer cell proliferation and thus is not selected for during cancer evolution.

In addition to its function in DNA repair, PARP1 also acts as a cofactor in regulating the expression of genes involved in many other biological processes [5]. Therefore, it is possible that altered expression of genes other than those involved in DNA repair and redox regulation may also contribute to the therapeutic effect of PARP inhibition. Identification and characterization of additional pathways that are regulated by PARP1 and targetable by PARP inhibition may help to expand the application of PARP inhibitors to cancers that are not deficient in HRR.

## 4 Materials and methods

### 4.1 Tissue samples

#### 4.1.1 The tissue samples for western blot

High-grade serous ovarian carcinoma (HGSOC) and fallopian tube tissues were collected in Qilu Hospital of China from April 2008 to July 2012. The HGSOC specimens were obtained from primary ovarian cancer patients receiving no surgery or chemotherapy previously. Fallopian tube tissues were from patients who receiveda total hysterectomy and bilateral salpingo-oophorectomy for uterine diseases or for benign neoplastic adnexal pathologic changes. All the fresh samples were obtained at surgery, immersed in RNAlater(Ambion) and stored at −80 °C. Ethics Committee of Shandong University approved the study and all participants gave written informed consent.

#### 4.1.2 Tissue microarray for immunohistochemistry

Fresh primary ovarian carcinoma tissues were obtained from chemotherapy naïve ovarian cancer after resection at the Prentice Women's Hospital of Northwestern University. Prior to surgery, written informed consent for tissue acquisition was obtained. All tumors were collected and engrafted within 2 h post resection. The use of patient tissues was approved by the Institutional Review Board for Human Research (IRB) at Northwestern University and all patients provided written consent for the use of their tissue for research purposes.

### 4.2 Cell culture

FTE-187 (immortalized normal human fallopian tube epithelial cell line) cell line was as described [29]. SKOV3, HEY and A2780 cells were as previously described [30]. HO8910, HO8910 P.M. and OVCAR3 cells were purchased from Shanghai Cell Bank, Chinese Academy of Sciences (Shanghai,China). HEY and A2780 cells were cultured in DMEM medium (Gibco, Invitrogen). SKOV3 cells were cultured in McCoy's 5A medium. FTE-187 cells were maintained in cell culture medium consisting of 1:1 Medium199 (Sigma-Aldrich) and MCDB105 medium (Sigma- Aldrich). HO8910, OVCAR3 and HO8910 P.M. cells were cultured in RPMI 1640 medium (Gibco, Invitrogen). All media contained 10% FBS (GIBCO, Invitrogen), 100 µg/mL penicillin, and 100 µg/mL streptomycin. All cells were cultured in a humidified atmosphere of 5% CO_2_ at 37 °C.

### 4.3 Chemicals and antibodies

PJ-34 and GKT137831(GKT) were purchased from Selleck. NAC was purchased from Beyotime Institute of Biotechnology (China). Dioleoyl-snglycero-3-phosphocholine (DOPC)was purchased from Aladdin (China). MitoSOX™ Red Mitochondrial Superoxide Indicator was from Life Technologies. MTT (3-[4, 5-dimethyl-2-yl]-2, 5-diphenyl tetrazolium bromide) and all other chemicals were from Sigma Chemical. The antibodies against 8-OHdG (sc-393871) and β -actin (sc-69879) were acquired from Santa Cruz Biotechnology. The antibodies against PARP1(9532) and Ki-67 (9129S) were purchased from Cell Signaling Technology. The antibodies against NOX1 (ab55831) and NOX4 (ab133303) were from Abcam. Anti-γ-H2AX (Ser139) was purchased from Upstate Biotechnology Inc. Anti-GAPDH(60004-1-Ig) was from Proteintech.

### 4.4 Western blot analysis

Cells were harvested and lysed on ice for 30 min in lysis buffer (Beyotime, China). The protein concentration was determined by the BCA assay kit (Beyotime, China). 30–50 μg protein samples were separated by SDS-PAGE (6–12%) and electro-transferred onto PVDF membrane. The membrane was blocked with 10% skim milk and incubated with specific primary antibodies at 4 °C for overnight. Proteins of interest were detected with appropriate horseradish peroxidase-conjugated secondary antibodies and developed using ECL kit (Thermo). The protein levels were normalized by β –actin or GAPDH.

### 4.5 RT-PCR

Total RNA was extracted from frozen tissues or cultured cells using TRIzol reagent (Invitrogen), and was reverse transcribed using reverse transcriptase (TOYOBO). Quantitative real-time PCR analysis was performed on a Roche LightCycler® 480 System using SYBR GREEN mix (TOYOBO). Human glyceraldehyde 3-phosphate dehydrogenase (GAPDH) was amplified as an internal control. The levels of PARP1 and GAPDH mRNA were measured by the SYBR Green I assay. PARP1 was amplified by using the primers with the sequence 5’-CTCTCCAATCGCTTCTACAC-3’ (forward) and 5’-GTTGTCTAGCATCTCCACCT-3’ (reverse). The GAPDH primers were 5’-CAGAACATCATCCCTGCCTCTAC-3’ (forward) and 5’-TTGAAGTCAGAGGAGACCAC

CTG-3’ (reverse). The samples were loaded in quadruple, and the results of each sample were normalized to GAPDH.

### 4.6 Cell cycle analysis

Cells were harvested at various time points after PJ-34 treatment, washed once with cold PBS, and then fixed in 70% cold ethanol at −20 ℃ overnight. The fixed cells were washed with PBS once and then stained with 50 μg/mL propidium iodide and treated with RNase A (100 μg/mL) together for 30 min at room temperature. Cell cycle analysis was performed on a FACScan flow cytometer (BD FACSCaliburTM). 10,000 cells were harvested for each sample. Finally, the fraction of cells in G1 phase, S phase and G2/M phase was determined using ModFit software.

### 4.7 Apoptosis analysis

Ovarian cancer cells, treated with different doses of PJ-34, were harvested and washed once in cold PBS, and then stained with Alexa Fluor® 488 annexin V and Propidium iodide (Alexa Fluor® 488 annexin V/Dead Cell Apoptosis Kit with Alexa® Fluor 488 annexin V and PI for Flow Cytometry, invitrogen) and analyzed by flow cytometry using 488 nm excitation. Finally, the fraction of early apoptotic cells was determined with FCS Express V3 software.

### 4.8 Measurement of ROS

ROS generation was measured using oxidation sensitive fluorescent probe (DCFH-DA) according to the manufacturer's protocols (Beyotime, China). Cells were treated with PJ-34 in the absence or presence of GKT for the indicated times. After the incubation, the cells were harvested and then stained with 10 μM DCFH-DA probe at 37 °C for 20 min. Cells were washed three times with PBS, and the induction of ROS was examined by flow cytometry. In all experiments, 10,000 viable cells were analyzed.

### 4.9 Clonogenic assay

Single-cell suspensions were generated for each cell line and specified numbers of cells were seeded into six-well tissue culture plates. Then cells were exposed to different doses of PJ-34, NAC, or GKT for two weeks. Colonies were stained with crystal violet. Colonies of greater than 50 cells were counted to determine the surviving fraction. The data presented are the mean ± standard error (SE) and represent three independent experiments.

### 4.10 Immunofluorescence staining of DNA damage markers

Immunofluorescence staining was carried out as described previously [31]. Briefly, cells grown on coverslips were fixed in 4% paraformaldehyde for 10 min. The cells were then permeabilized in 0.2% Triton X-100 for 10 min, and blocked in 10% normal goat serum overnight at 4 °C. The coverslips were incubated with anti-phospho-H2AX,8-OHdG antibody overnight at 4 ℃,washed in PBS, and incubated with TRITC-conjugated Goat anti-mouse secondary antibody (Jackson Immuno Research Laboratories, West Grove, PA) for 1 h at room temperature. Cells were washed in PBS three times and counterstained with DAPI. Fluorescence images were captured under a fluorescence microscope.

### 4.11 RNA interference

All small interfering RNAs (siRNAs) were purchased from Sigma - Aldrich. The siRNA targeting human NOX1 (Sense strand 5′-UCUGCUCUCUGCUUGAAUUdTdT-3′ and antisense strand 5’-AAUUCAAGCAGAGAGCAGAdTdT-3’) and NOX4 (Sense strand 5’-CGAAAGACUUUACAGGUAUdTdT-3’ and antisense strand 5’-AUACCUGUAAAGUCU)UUCGdTdT-3’ were applied as a mixture at a total final concentration of 100 nM. A non-silencing scramble RNA duplex was used as the negative control (Sense strand 5’-UUCUCCCGAACGUGUCACGUTTdTdT −3’ and antisense strand 5’-ACGUGACACGUU

CGGAGAATTdTdT-3’). A2780 and HO8910 cells were transfected with siRNAs using Lipofectamine 2000 (Invitrogen, CA). NOX1 and NOX4 protein levels were determined by Western blotting analysis.

### 4.12 Liposomal siRNA preparation

For in vivo delivery, siRNA was incorporated into dioleoyl-snglycero-3-phosphocholine (DOPC). DOPC and siRNA were mixed in the presence of excess tertiary butanol at a ratio of 1:10 (w/w) siRNA/DOPC. Before in vivo administration, the preparation was hydrated with normal 0.9% saline (100 µL per mice) for i.v.(intravenous injection).

### 4.13 Tumor xenografts

Six-weeks-old female nude mice were purchased from Beijing Experimental Animal Center and kept in pathogen-free conditions and handled in accordance with the requirements of the Guideline for Animal Experiments. Animals were subcutaneously inoculated with 1 × 10^6^ A2780 cells (suspended in 200 µL PBS), seven days later the animals were randomly divided into four groups and treated with either vehicle control (DMSO), PJ-34 alone (10 mg/kg given daily by intraperitoneal injection), GKT alone (12 mg/kg given daily by intraperitoneal injection), or 10 mg/kg PJ-34 plus 12 mg/kg GKT (given daily by intraperitoneal injection). Nude mice were also subcutaneously inoculated with 1 × 10^6^ A2780 shNeg or A2780 shPARP1 cells (suspended in 200 µL PBS),and seven days later, were subjected to liposomal siRNA treatments. Each mice received 150 μg/kg (equivalent of 4 μg/mice) non-silencing control siRNA or NOX1/4 siRNA once every three days (i.v. into the tail vein in 100 µL saline) for two weeks. Tumor growth was monitored with a caliper, and tumor volume was calculated according to the formula V = maximal diameter × perpendicular diameter ^2^. After the last treatment on day 22, tumors were removed and weighed.

### 4.14 Immunohistochemistry of tumor xenografts

After deparaffinization and rehydration, the sections were boiled in citrate sodium buffer for 15 min for antigen recovery, and immersed in 3% H_2_O_2_ for 10 min to quench endogenous peroxidase. Sections were then blocked with 10% serum at 37 °C for 1 h. The primary antibodies (Ki-67, CST, 1:200 dilution; 8-OHdG, Santa, 1:80 dilution) were added to the sections and incubated overnight at 4 °C. After washing, the sections were coated with a horseradish peroxidase (HRP) conjugated second antibody (Jackson ImmunoResearch; 1:200 dilution) and then incubated at 37 °C for 1 h. DAB was used to visualize immunoreactions.

### 4.15 Statistical analysis

The software SPSS V20.0 was used for statistical analysis. All data were presented as means and standard errors. Student's *t*-test and one-way ANOVA analysis were used to determine significance. *P* < 0.05 was considered statistically significant. Statistical significance was also taken as *P < 0.05, **P < 0.01 and ***P < 0.001.
