# Supplementary figures and images for: Increased oxidative stress mediates the antitumor effect of PARP inhibition in ovarian cancer
Source: Redox Biol. 2018 Mar 30;17:99–111. doi: 10.1016/j.redox.2018.03.016 (PMC6006521; doi:10.1016/j.redox.2018.03.016)

Figure S1

A

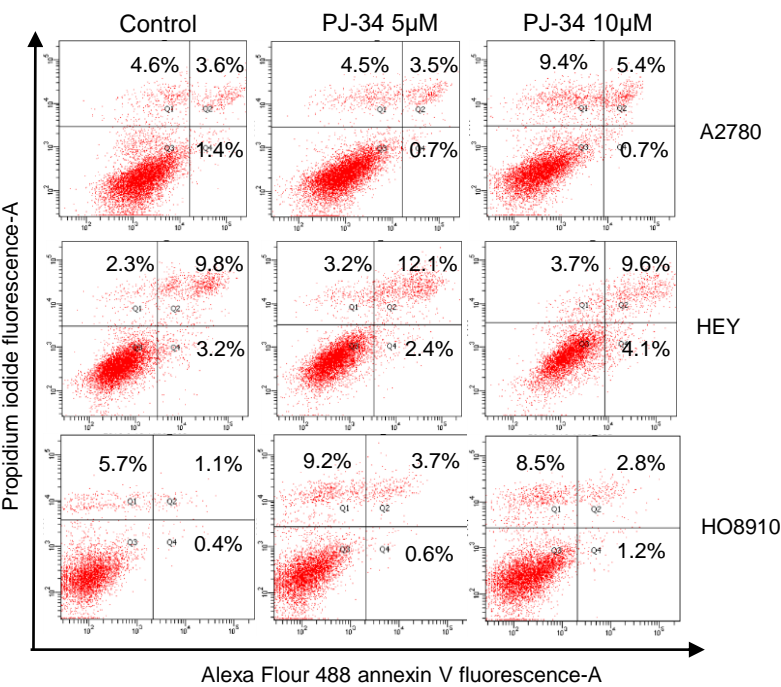

# Figure S2

**A**

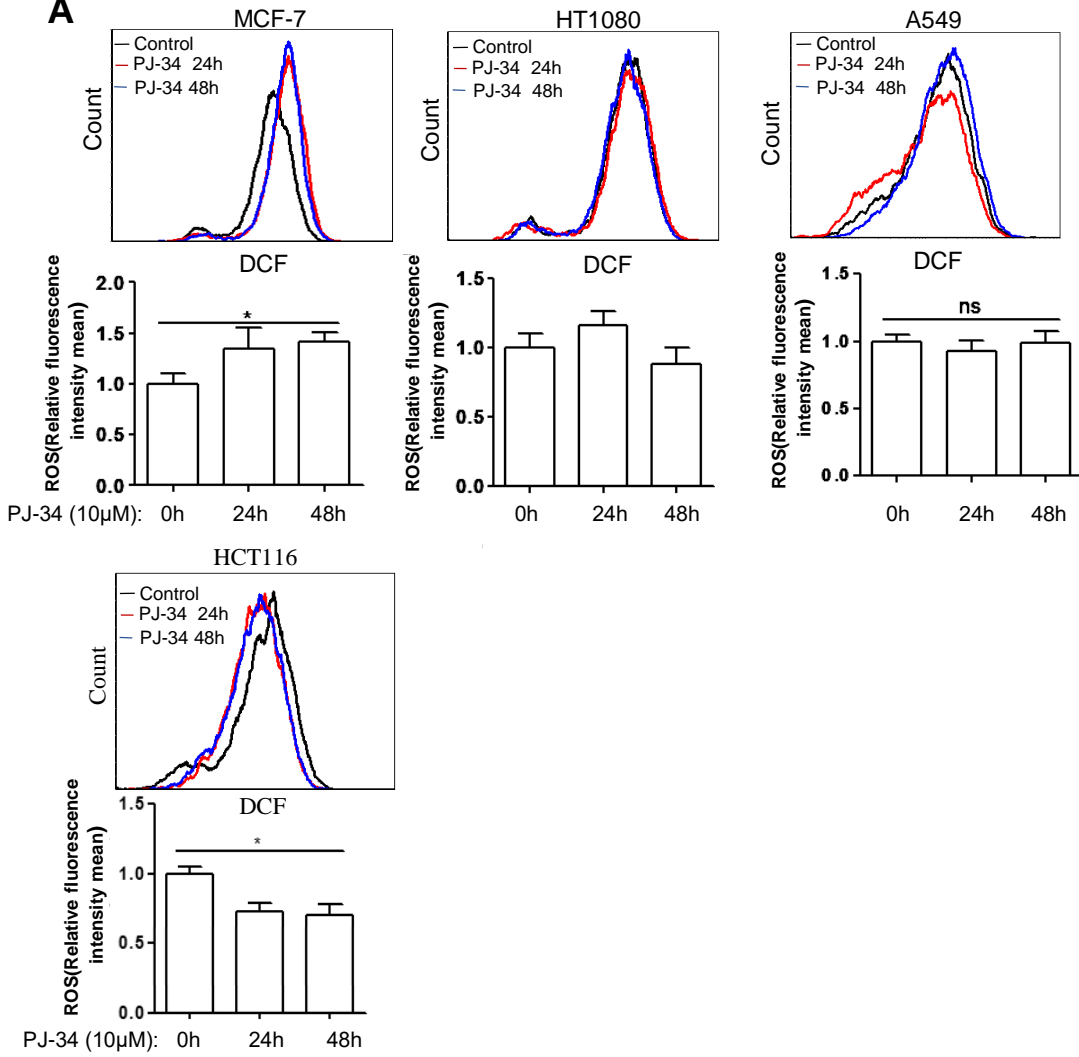

# Figure S3

**A**

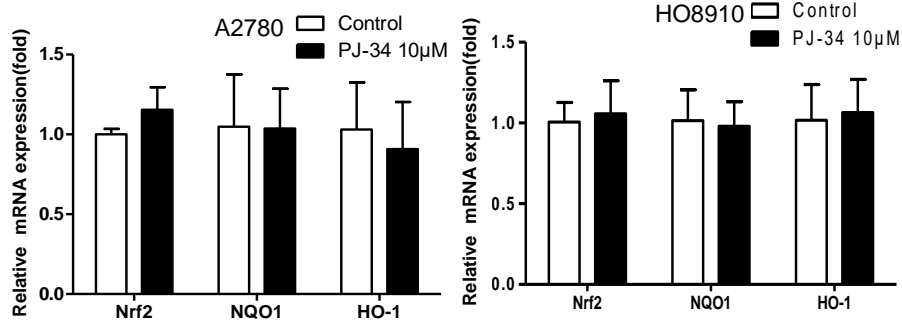

**B**

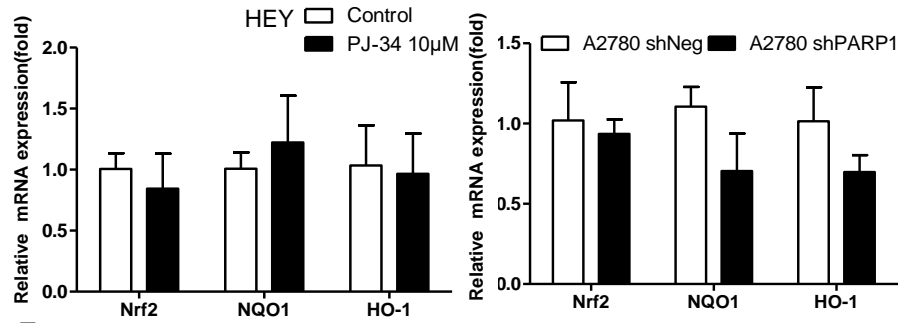

**C**

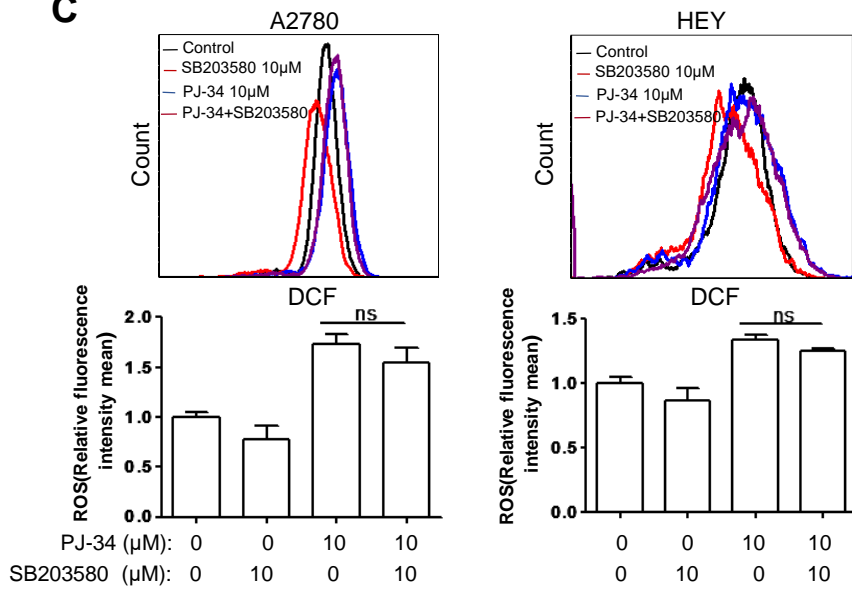

Supplement: Supplementary file 1 — Supplementary material [file mmc1.pdf]
